# Carbon Dots and Mitochondria—Advances in Targeting, Imaging, and Therapeutics

**DOI:** 10.3390/ijms27031469

**Published:** 2026-02-02

**Authors:** Aasia Bibi, Daniela De Benedictis, Giuseppe Capitanio, Alessandra Gabriele, Amer Ahmed, Mariapompea Cutroneo, Lorenzo Torrisi, Daniela Manno, Antonio Serra, Domenico De Rasmo, Anna Signorile

**Affiliations:** 1 Department of Translational Biomedicine and Neuroscience, University of Bari Aldo Moro, 70124 Bari, Italy; aasiabibi250@gmail.com (A.B.); debenedictis.d0@gmail.com (D.D.B.); giuseppe.capitanio@uniba.it (G.C.); 2 CEDAD—Centro di Fisica Applicata Datazione e Diagnostica—Dipartimento di Matematica e Fisica “E. De Giorgi”, Università del Salento, Via Arnesano, 73100 Lecce, Italy; alessandra.gabriele@unisalento.it (A.G.);; 3 Department of Biosciences, Biotechnologies and Environment, University of Bari Aldo Moro, 70125 Bari, Italy; 4 Dipartimento MIFT, Università di Messina, Viale F.S. d’Alcontres 31, 98166 Messina, Italylorenzo.torrisi@unime.it (L.T.); 5 CNR-Institute of Biomembranes, Bioenergetics and Molecular Biotechnologies, 70124 Bari, Italy; d.derasmo@ibiom.cnr.it

**Keywords:** CDs (carbon dots), mitochondria, imaging, therapeutics

## Abstract

Carbon dots (CDs), a class of fluorescent nanomaterials, have emerged as powerful tools for biological applications, particularly in the targeting, imaging, and therapeutic modulation of mitochondria. Due to their small size, simplicity of synthesis, biocompatibility, and tunable optical properties, CDs can be engineered to selectively accumulate in mitochondria, enabling real-time imaging of mitochondrial function and dynamics in live cells. Moreover, their ability to carry therapeutic agents, such as antioxidants, drugs, and gene delivery vectors, offers potential in treating mitochondrial dysfunction, which is central to various diseases, including neurodegenerative disorders, cancer, and metabolic diseases. Recent advancements in surface functionalization have enhanced mitochondrial targeting and specificity, while ongoing research aims to optimize the safety, efficiency, and clinical translation of CDs for therapeutic applications. This review highlights the latest developments in the use of carbon dots for mitochondrial imaging, therapeutic delivery, and disease intervention, offering promising avenues for future research and clinical applications.

## 1. Introduction

Carbon dots (CDs) are a class of fluorescent nanomaterials that have gained significant attention in recent years due to their unique optical properties, biocompatibility, and ease of synthesis [1,2,3].

Structurally, CDs may consist of nanoscale sp^2^-hybridized graphitic domains, sp^3^-hybridized carbon regions, or other carbonaceous arrangements, forming a heterogeneous and often partially disordered architecture [4,5].

The photoluminescence of CDs originates from the interplay of multiple emissive centers, including quantum-confined sp^2^ domains, surface defect states, and small molecular fluorophores that are generated, trapped, or retained during the synthesis process [6]. This multifaceted emission mechanism confers highly tunable and environment-sensitive optical properties, enabling excitation- and size-dependent fluorescence across a broad spectral range. In addition, CDs exhibit high chemical and photostability, intrinsic water solubility, and generally low biological toxicity, making them highly suitable for a variety of biomedical applications, including bioimaging, biosensing, targeted drug delivery, and gene therapy [7,8,9].

Collectively, these chemical, physical, and biological features allow CDs to overcome many limitations associated with conventional fluorescent probes for live-cell imaging, such as photobleaching, cytotoxicity, and limited functionalization potential [10].

In addition, their ability to be engineered with specific functional groups that specifically target cellular organelles has opened exciting opportunities for organelle monitoring and for studying their modulation in several physiological and pathological conditions [11,12,13,14].

In this context, CDs can be designed to selectively accumulate within mitochondria, enabling high-resolution and real-time imaging of mitochondrial morphology, membrane potential, and reactive oxygen species (ROS) levels [15,16,17].

Mitochondria, often referred to as the “powerhouses” of the cell, play a central role in energy production through oxidative phosphorylation. Beyond this, they regulate apoptosis, calcium homeostasis, and ROS production [18,19,20].

Mitochondria also influence cellular signalling and are integral to processes such as aging, immunity, and metabolism. Given their essential role in cellular homeostasis, mitochondrial dysfunction profoundly affects cell health and contributes to the development of several diseases, including neurodegenerative disorders (e.g., Alzheimer’s and Parkinson’s), cardiovascular diseases, diabetes, and cancer [21,22,23,24].

Therefore, understanding and monitoring mitochondrial health in living organisms is essential for disease diagnosis, prognosis, and therapeutic intervention [25,26].

The recognition of mitochondrial dysfunction as a key factor in numerous pathological conditions has driven the development of advanced tools to analyze mitochondrial dynamics and to design therapies aimed at restoring normal cellular function. The non-invasive and versatile nature of CD-based imaging allows the investigation of mitochondrial behavior in live cells under various physiological and pathological conditions, providing valuable insights into mitochondrial dysfunction [21,27].

Moreover, the surface of CDs can be modified to carry therapeutic agents such as antioxidants, drugs, or genetic material, allowing their direct delivery to mitochondria. This capability offers promising applications for the treatment of mitochondrial-related diseases [28].

For instance, antioxidant-loaded CDs can reduce mitochondrial oxidative stress, while those functionalized with gene-delivery vectors can target specific mitochondrial genes to correct defects or modulate function, also for detection and treatment of cancer [29,30].

Drug-loaded CDs targeting mitochondrial pathways involved in apoptosis or energy metabolism also represent powerful tools for therapeutic intervention. Thus, the combination of fluorescent properties, mitochondrial targeting, and drug delivery capacity makes CDs valuable instruments for understanding mitochondrial function, diagnosing mitochondrial diseases, and developing new therapeutic strategies [31,32].

Despite these promising results, several challenges remain. These include optimizing targeting efficiency to ensure specific mitochondrial localization, assessing long-term biocompatibility and potential toxicity, and translating laboratory findings into clinical applications. Furthermore, achieving scalable and reproducible synthesis is essential for future biomedical use [16,33,34].

This review provides an overview of recent advances in carbon dot research, focusing on their applications in mitochondrial targeting, imaging, and therapeutics. It discusses their use as mitochondrial probes for real-time imaging, their potential for targeted drug and gene delivery, and their role in mitochondrial protection and repair. Finally, it highlights current challenges and future perspectives, emphasizing the potential of CDs to revolutionize mitochondrial research and precision medicine.

## 2. Carbon Dots (CDs): What Are They?

**Figure 1:**
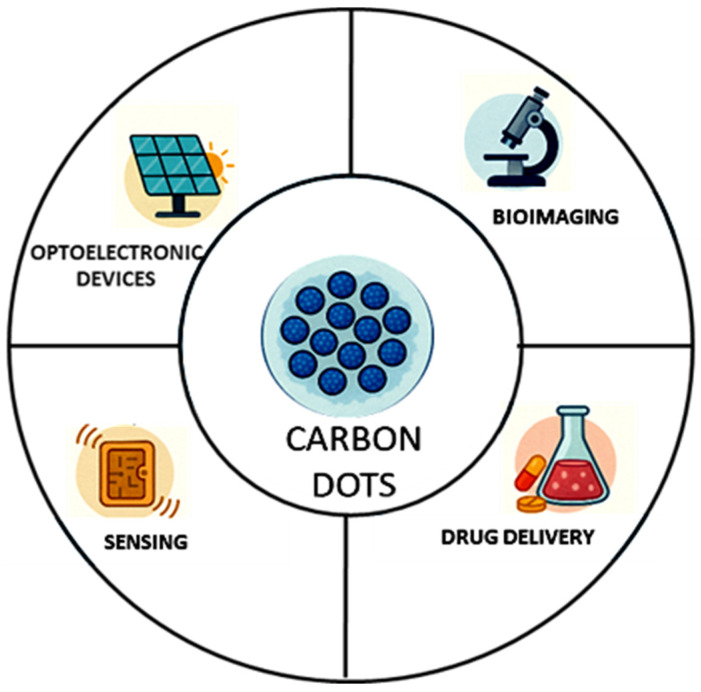
Biomedical and technological applications of CDs.

Carbon dots (CDs) are a class of carbon-based nanomaterials that exhibit unique optical, electrical, and chemical properties. These nanoscale materials, typically ranging from 1 to 10 nanometers in size, and their tunable physicochemical features are dictated by the synthesis methodology and precursor materials. Discovered in 2004, CDs were found to possess unexpected fluorescent properties for carbon-based materials. The nanoscale dimensions of CDs enable efficient penetration into biological systems and promote strong cellular interactions, making them highly attractive for nanomedical applications such as cancer diagnosis, drug delivery and biosensing [35] (Figure 1).

CDs can act as nanocarriers for drugs to target sites precisely, improving both therapeutic efficiency and safety. Carbon dots (CDs) are promising materials for non-invasive diagnostics and biological tracking, and they can be incorporated into solar panels to enhance the efficiency of light-to-electric energy conversion [36].

The ability of CDs to change their luminescence (the way they emit light) when they come into contact with specific molecules or ions they could represent excellent “detectors” [37].

Since then, research on these nanomaterials has rapidly expanded, owing to their outstanding optical characteristics, ease of synthesis, and excellent biocompatibility. They are also referred to as carbon quantum dots (CQDs) or carbon nanoparticles in some contexts [38,39].

**Figure 2:**
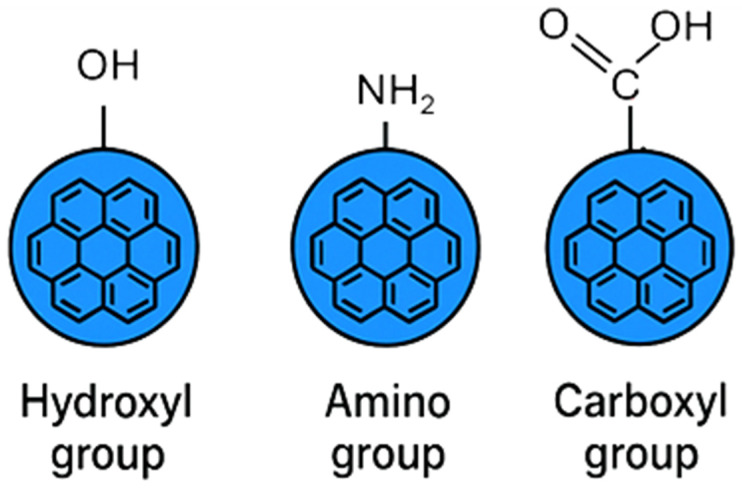
General structure of carbon dots.

Typically CDs are composed of a carbon core and surface region functionalized by different types of molecules, and can vary depending on the synthesis method. The carbon core consists of sp^2^-hybridized graphitic domain embedded within a sp^3^-hybridized amorphous carbon matrix. This hybrid structure exhibits different degrees of crystallinity, resulting in a different relative proportion of crystalline to amorphous regions. The surface of CDs typically contains oxygen-functional groups such as hydroxyl (–OH), carboxyl (–COOH), and amine (–NH_2_) groups [4,40,41] (Figure 2).

The carbon core is the major responsible for the electronic and fluorescent properties. The fluorescence of CDs is their most widely used property for bioimaging and sensing. Their light emission is complex because it can come from the tiny, quantum-confined carbon core, chemical molecular groups bonded to the surface, and small fluorescent molecules attached to or trapped inside the dot [42,43,44].

This complexity is useful, because it allows to adjust absorption and emission light of CDs. Their fluorescence can be tuned across a broad spectrum (from ultraviolet to visible light) depending on size, surface modification, and synthesis method [45,46,47,48], and understanding how size, structure, and photophysical behavior relate to one another is key to designing CDs that work effectively for biomedical applications (bioimaging, biosensing, theranostics) [49,50,51].

Schematic representation of common surface functional groups on carbon dots, including hydroxyl (–OH), amino (–NH_2_), and carboxyl (–COOH) groups, which play a key role in determining their solubility, surface reactivity, and biological interactions.

CDs exhibit remarkable chemical and photostability, resisting degradation under prolonged light exposure and harsh conditions, which supports their use in long-term bioimaging and sensing. They are generally biocompatible and show low toxicity, making them suitable for biological use. Their intrinsic water solubility further facilitates their integration into biomedical environments that require aqueous conditions. In addition, the ability to functionalize CDs with biocompatible surface groups not only improves their interaction with biological tissues but also broadens their range of potential applications. The possibility to control these fundamental nanoscale properties; size, structure, and photophysics is the key point in designing targeted CDs for advanced biomedical applications The possibility to control these fundamental nanoscale properties; size, structure, and photophysics is the key point in designing targeted CDs for advanced biomedical applications [45,46,47,48].

## 3. Synthesis of Carbon Dots

**Figure 3:**
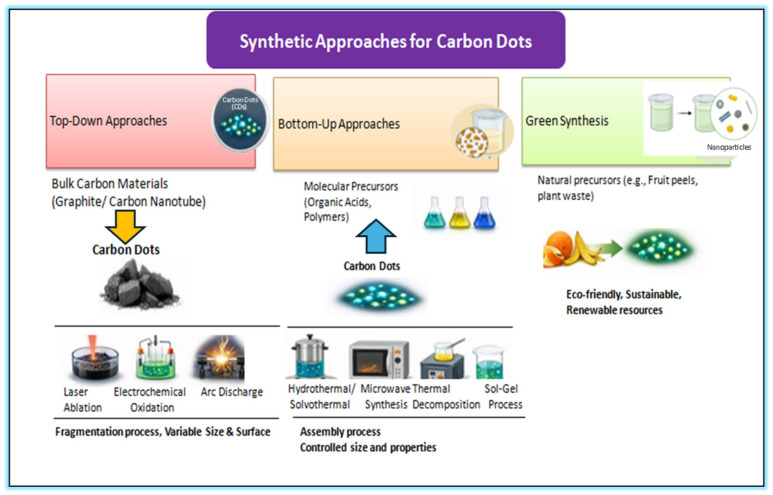
Synthetic approaches for carbon dots.

Carbon dots (CDs) can be synthesized through various methods, each influencing their final physicochemical properties. The two main synthesis strategies are top-down and bottom-up approaches (Figure 3).

In the top-down synthesis, larger carbon-based materials (e.g., graphite, carbon nanotubes) are broken down into smaller nanoscale fragments using physical or chemical processes such as laser ablation, electrochemical oxidation, or acidic oxidation. While this method enables the direct conversion of bulk carbon into nanosized dots, it is often less controllable and may produce heterogeneous mixtures of particles with variable sizes and surface properties [52,53,54].

In particular, the laser carbon ablation permits the synthesis of CDs directly into PBS solutions, and isotonic physiological solutions based on NaCl and glucose, maintaining high sterility and stabilizing the biocompatible liquids, ready to be injected into cell cultures. The photoluminescence emission of the synthesized CDs, in terms of photon energy and yield, can be controlled by the laser parameters, solution composition, and laser irradiation conditions [55,56].

Overview of the main synthetic strategies employed for the preparation of carbon dots (CDs). Three major routes are illustrated for the preparation of CDs: (i) top-down approaches, in which bulk carbon materials such as graphite or carbon nanotubes are fragmented into nanoscale CDs via laser ablation, electrochemical oxidation, or arc discharge; (ii) bottom-up approaches, where molecular precursors including organic acids and polymers are assembled into CDs through hydrothermal/solvothermal treatment, microwave-assisted synthesis, or sol–gel processes, allowing controlled tuning of particle size and surface properties; (iii) and green synthesis, which utilizes natural and renewable precursors such as fruit peels and plant waste to produce eco-friendly and sustainable CDs. These complementary methods enable versatile control over the structural and optical properties of carbon dots for diverse applications.

Conversely, the bottom-up synthesis involves assembling CDs from small molecular precursors, such as organic acids or polymers. Common techniques include hydrothermal or solvothermal treatment, microwave-assisted synthesis, and thermal decomposition. This approach offers greater control over particle size, morphology, and surface functionalities, typically yielding high-quality and more stable carbon dots compared to top-down methods [56,57].

An emerging alternative is the green synthesis of CDs, which employs natural precursors such as plant extracts or biowaste materials (e.g., fruit peels, plant fibers). This environmentally friendly and sustainable route provides a renewable and cost-effective source of carbon dots suitable for diverse applications [54,58,59] (Figure 3).

### 3.1. Top-Down Synthesis

The top-down method involves breaking down larger carbon-based materials (such as graphite or carbon nanotubes) into smaller particles or nanostructures. This approach is generally simpler and can be used to produce carbon dots from bulk materials [60,61].

However, the size and surface properties of the resulting carbon dots may be more difficult to control. For this approach several techniques can be used such as laser ablation, electrochemical oxidation, and arc discharge. In the laser ablation approach, a laser is focused on a carbon target (e.g., graphite, carbon nanotubes, graphite oxide, graphene, and others) in a liquid or gas medium. The high-energy laser pulses break down the larger carbon materials, leading to the formation of carbon nanoparticles (carbon dots) [55,62,63,64].

The carbon target is typically placed in a liquid medium like water, PBS or physiological solutions, or an organic solvent. The laser is then applied, causing the carbon to vaporize, ionize, and condense into small carbon nanoparticles [65,66,67].

Recent studies have demonstrated the synthesis of CDs directly from commercially available charcoal using a continuous-wave laser diode (450 nm) in PBS solution, without any chemical reagents, producing biocompatible CDs with an average size of ~10 nm and blue luminescence at 480 nm and 520 nm. This approach allows embedding the CDs into polycaprolactone (PCL) scaffolds, preserving luminescence and enabling potential applications in tissue engineering and bone scaffolds [55]. Laser ablation has the advantage of precise control over size, and morphology can be achieved by adjusting the laser power and ablation time [65,66].

The CDs functionalization depends on the used laser parameters, liquid, target composition, and irradiation conditions. The method is highly reproducible and scalable for larger quantities. However, several challenges are associated with this method, including the generation of a mixture of various sizes, requiring additional purification steps. Also, the use of lasers can be costly and may not be suitable for all types of precursor materials [60,68].

Synthesis of CDs by electrochemical oxidation consists of oxidation of larger carbon materials (such as graphite or carbon nanotubes) using an electrochemical cell, often in a solution containing salts or acids. The oxidation process results in the breaking down of the bulk carbon into smaller, functionalized nanoparticles. A carbon material is used as the anode in an electrochemical cell, and the oxidation is carried out in the presence of an electrolyte. The process produces carbon nanoparticles that are then collected and purified. This method is relatively simple and cost-effective and can be performed under mild conditions. However, difficulty in controlling the size distribution and surface chemistry, and optimization requirements to prevent over-oxidation and the formation of undesired products are the major limitations [64,69,70,71,72].

Arc Discharge is a method that uses an electric arc between two carbon electrodes (such as graphite rods) in the presence of a solvent or gas. The high-energy discharge causes the carbon material to vaporize and then condense into nanoscale carbon particles. The carbon material is vaporized by an electric arc in a controlled environment, and the vapor condenses into nanoparticles. In this method, high-quality carbon dots can be produced and are useful for producing high-purity carbon nanoparticles. However, this method requires specialized equipment and is less scalable compared to other methods, and can result in non-uniform particle size and a mixture of carbon-based materials [62,64,73,74] (Figure 3).

### 3.2. Bottom-Up Synthesis

The bottom-up synthesis approach involves building carbon dots from smaller precursor molecules, such as organic compounds or simple carbon-based materials. This method allows for greater control over the properties of the resulting carbon dots, such as size, surface functionality, and fluorescence [62,64].

Several techniques are usually employed in this approach, including Hydrothermal/Solvothermal Synthesis, Microwave-Assisted Synthesis, Thermal Decomposition (Pyrolysis), and Sol–gel Process.

Hydrothermal/Solvothermal Synthesis involves heating a mixture of organic precursor materials in a sealed vessel, typically at high pressure and temperature, to form carbon dots. The solvents used may include water (hydrothermal) or organic solvents (solvothermal). Organic compounds (such as citric acid, glucose, or other carbohydrates) are mixed with a solvent and heated in a high-pressure autoclave. The heat and pressure promote the carbonization and condensation of the organic precursor, leading to the formation of carbon dots. It is simple and cost-effective and enables high control over size and surface chemistry. It is used to produce carbon dots from a wide range of organic precursors. Though the reaction conditions (temperature, pressure, precursor concentration) need to be carefully optimized, and high temperatures may lead to the formation of undesirable byproducts [75,76,77].

Microwave-Assisted Synthesis utilizes microwave radiation to rapidly heat the precursor materials in a solvent, allowing for the efficient formation of carbon dots. Organic precursors, such as glucose, citric acid, or other small organic molecules, are mixed with water or an organic solvent and exposed to microwave radiation. The microwave energy accelerates the reaction, leading to the formation of carbon dots. It is fast and efficient compared to traditional heating methods and provides better control over the size and uniformity of the carbon dots. Challenges include the requirement for a microwave reactor, which may limit scalability and control over the size and surface characteristics of the carbon dots [57,78,79,80].

Thermal decomposition involves heating organic precursors to high temperatures in the absence of oxygen, promoting carbonization and the formation of carbon dots. A precursor material (such as glucose, citric acid, or other organic compounds) is heated at high temperatures (typically 200–300 °C) under an inert atmosphere (e.g., nitrogen or argon). The organic material undergoes pyrolysis, breaking down into carbon dots. It is simple and cost-effective and allows for the synthesis of carbon dots from inexpensive carbon-rich precursors. Though it requires careful temperature control to avoid over-carbonization, which may lead to irregularly shaped particles formation [81,82,83].

The sol–gel process involves the polymerization of precursor materials into a gel phase, which is then heated to form carbon dots. A precursor solution (such as a mixture of citric acid and an amine or alcohol) is prepared, and the solvent is evaporated under controlled conditions. The gel that forms is then heated to produce carbon dots. It is suitable for large-scale production of carbon dots and can produce carbon dots with good uniformity and high surface functionality. However, it requires careful control of reaction conditions to avoid undesired products, and the synthesis time may be longer compared to other methods [84,85,86,87] (Figure 3).

#### Green Synthesis

Green synthesis is a subtype of bottom-up synthesis. This approach is based on the principles of sustainability and environmental safety It uses natural, biodegradable, or non-toxic precursors (e.g., fruits, vegetables, plant extracts, biomass) and environmentally friendly conditions (e.g., water as solvent, moderate temperatures, minimal use of toxic chemicals) [54,88,89,90].

These materials are subjected to heat, microwave, or hydrothermal treatment to produce carbon dots. It is environmentally friendly and sustainable, and utilizes renewable and cost-effective raw materials. However, the synthesis may result in a less controlled product in terms of size and fluorescence. Extracts from natural materials may contain impurities or residual biomolecules, which may affect the purity, reproducibility, and optical performance of the resulting carbon dots [91,92,93] (Figure 3).

## 4. Carbon Dots and Biological Applications

CDs are versatile nanomaterials in biological research due to their small size, excellent biocompatibility, and tunable fluorescence. They are widely used for high-resolution bioimaging, intracellular tracking, and sensitive detection of biomolecules such as ions, reactive oxygen species, and nucleic acids. Additionally, CDs can serve as carriers for targeted drug delivery, enabling theranostic applications that combine imaging with therapy, and have shown potential in photodynamic and photothermal treatments. These properties make CDs a powerful tool for studying cellular processes and developing novel diagnostic and therapeutic strategies [51,94].

### Cellular Interaction and Applications of Carbon Dots

The stable and bright fluorescence of CDs makes them excellent tools for bioimaging in live cells [15,95].

**Figure 4:**
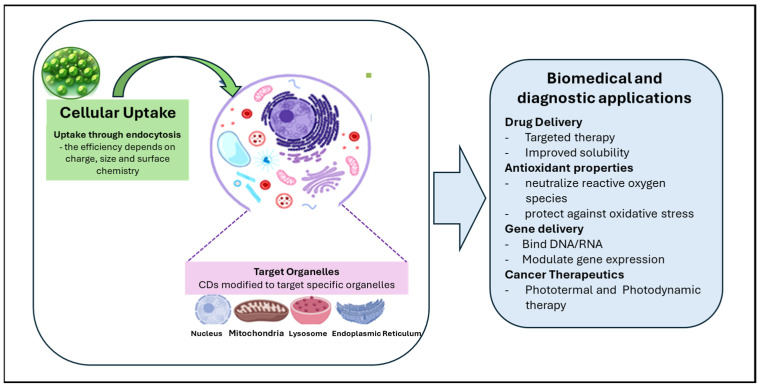
Cellular Interaction of CDs through Endocytosis; and Applications of CDs in Drug Delivery, Antioxidant Properties, Gene Delivery, Cancer Therapeutics; CDs target different cellular organelles, i.e., Nucleus (DNA), Mitochondria, Lysosome, Endoplasmic Reticulum.

Carbon dots (CDs) are usually taken up by cells through endocytosis, a natural process where the cell membrane surrounds and internalizes external particles. Their uptake depends on properties like size, surface charge, and surface chemistry—for example, positively charged CDs are often absorbed more efficiently because they interact with the negatively charged cell membrane [12] (Figure 4). Once inside the cell, CDs can act as fluorescent probes, allowing researchers to track and visualize cellular processes in real time [96,97].

CDs are also promising for drug delivery. Their surfaces can be modified with targeting molecules that help deliver drugs specifically to certain cells or tissues, increasing treatment efficiency and reducing unwanted side effects. In addition, CDs can carry hydrophobic drugs, improving their solubility and absorption in the body [98].

Some studies have shown that CDs also have antioxidant properties, meaning they can neutralize reactive oxygen species (ROS) inside cells. This helps protect cells from oxidative stress, which is linked to inflammation, aging, and many diseases [99,100]. Besides delivering drugs, CDs can also serve as gene carriers. They can bind and protect genetic material, such as DNA or RNA, and help it enter cells to promote gene expression [9,101].

Their biocompatibility and low toxicity also make them suitable for in vivo cell tracking, allowing researchers to follow cell movement and behavior in living organisms [102].

In cancer research, CDs show potential in photothermal and photodynamic therapies. When exposed to light, they can either generate heat or produce reactive oxygen species, both of which can help destroy cancer cells in a controlled way [103,104].

CDs are able to target specific organelles representing an important tool to obtain valuable information for understanding the processes involved in the functions/dysfunction of organelles. For example, in recent years, numerous CDs have been engineered to target the nucleus, mitochondria, and lysosomes, providing crucial insights into their cellular activities. Beyond imaging, CDs can penetrate the nucleus, especially when functionalized with specific peptides or positively charged groups, enabling DNA/RNA discrimination and drug delivery [15,105,106,107].

They can target mitochondria via lipophilic cations or intrinsic affinity, allowing ROS detection and monitoring of cancer cells. In lysosomes, CDs modified with lipophilic compounds can measure pH and detect analytes such as formaldehyde. In the endoplasmic reticulum, functionalized CDs report intracellular pH variations, while in the Golgi apparatus, cysteine-based CDs facilitate imaging and targeted drug delivery [14,108].

The inter-organelle migration of CDs highlights their potential for subcellular monitoring, diagnostics, and biomedical applications [109].

## 5. Mitochondria-Targeted Carbon Dots: Design and Applications

Mitochondria play a central role in cellular metabolism, signaling, and apoptosis, and their dysfunction is closely associated with a wide range of diseases, including cancer, neurodegeneration, and cardiovascular disorders. The ability to selectively visualize and modulate mitochondrial processes is therefore of great importance for both fundamental biological studies and biomedical applications. In this context, carbon dots (CDs) have attracted increasing attention as a new class of fluorescent nanomaterials for mitochondrial targeting, owing to their small size, excellent photostability, tunable surface chemistry, and generally favorable biocompatibility. By rational design of their surface properties and molecular composition, CDs can be engineered to preferentially accumulate in mitochondria, enabling high-resolution imaging and the development of mitochondria-centered diagnostic and therapeutic platforms.

### 5.1. Mitochondria in Health and Disease: An Overview

Mitochondria are membrane-bound organelles found in almost all eukaryotic cells. They are often referred to as the “powerhouses of the cell” because of their essential role in energy production. However, beyond generating ATP, mitochondria perform numerous other vital functions, including metabolic regulation, control of apoptosis, calcium homeostasis, and cellular signaling. When mitochondrial activity is impaired, the resulting mitochondrial dysfunction can have widespread effects on cellular and organismal health, contributing to disorders such as neurodegenerative, metabolic, cardiovascular diseases, and cancer [23,24,110,111,112].

Mitochondria are central to cellular energy metabolism. They produce adenosine triphosphate (ATP)—the main energy currency of the cell—through oxidative phosphorylation (OXPHOS), which occurs in the inner mitochondrial membrane. This process involves the electron transport chain (ETC), a sequence of protein complexes that transfer electrons and create a proton gradient used by ATP synthase to generate ATP [113,114,115].

In addition to OXPHOS, mitochondria regulate metabolism through several interconnected pathways, including the citric acid cycle (Krebs cycle), fatty acid β-oxidation, and amino acid metabolism. These processes convert diverse substrates into energy intermediates, sustaining cellular function and growth [114,116].

Beyond energy production, mitochondria are deeply involved in maintaining calcium homeostasis, absorbing and releasing calcium ions to regulate intracellular signaling [20,116,117]. They also play a key role in apoptosis releasing pro-apoptotic proteins such as cytochrome c, activating caspase enzymes and initiating the controlled elimination of compromised cells [19,118,119].

**Figure 5:**
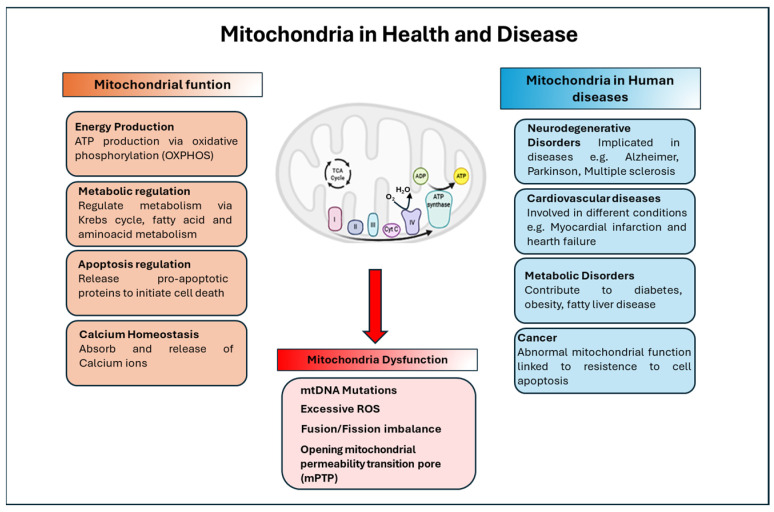
Mitochondria in Health and Disease.

Moreover, mitochondria are highly dynamic organelles, constantly undergoing fusion and fission to preserve their function, adapt to metabolic demands, and ensure proper distribution during cell division. This dynamic balance is essential for mitochondrial quality control and overall cellular homeostasis [118,120,121] (Figure 5).

Mitochondria regulate several function such as energy production, metabolic regulation, apoptosis and calcium homeostasis. Mitochondrial disfunctions are associated with excessive ROS production, alteration of dynamic (fusion/fission), opening mPTP. Mitochondrial dysfunctions are involved in several diseases.

Mitochondrial dysfunction can result from multiple mechanisms, including mutations in mitochondrial DNA (mtDNA), which disrupt oxidative phosphorylation; excessive generation of reactive oxygen species (ROS), leading to oxidative damage; imbalances in mitochondrial dynamics (fusion/fission defects); and opening of the mitochondrial permeability transition pore (mPTP), causing loss of membrane potential and release of apoptotic factors. Considering the involvement of mitochondria in these aspects, alterations in mitochondrial function are implicated in a wide spectrum of diseases such as neurodegenerative disorders, cardiovascular diseases, metabolic alteration, cancer and aging [22,24,110,112,122,123].

### 5.2. Carbon Dots for Mitochondrial Targeting

During oxidative phosphorylation, the generation of ATP is associated with a negative mitochondrial membrane potential. This electrochemical gradient facilitates the selective accumulation of lipophilic cations within mitochondria. Consequently, fluorophores conjugated to positively charged moieties such as methylpyridinium, triphenylphosphonium (TPP), and indolium cations have been widely employed for mitochondrial targeting due to their electrostatic attraction to the negatively charged inner membrane. These positively charged species preferentially accumulate within mitochondria due to the organelle’s highly negative transmembrane potential [124].

Owing to their delocalized positive charge and high lipophilicity, TPP-functionalized ligands have been extensively utilized for mitochondrial targeting [17,25,125,126,127].

**Figure 6:**
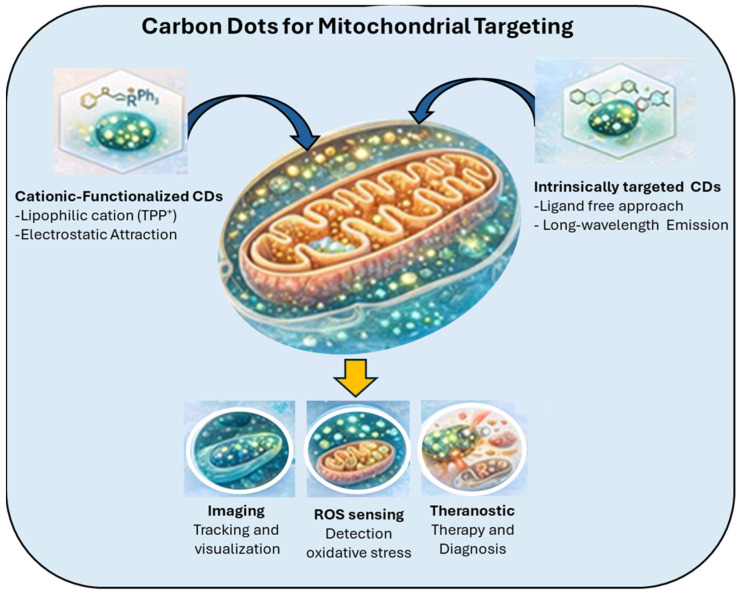
Carbon dots for mitochondrial targeting, imaging, ROS sensing, and theranostics.

These cations readily traverse the plasma membrane and the outer mitochondrial membrane due to their lipophilic nature, allowing them to accumulate several hundred-fold inside the mitochondria relative to the cytosol. This property has been extensively leveraged for both imaging and therapeutic applications, enabling researchers to monitor mitochondrial function, reactive oxygen species (ROS) generation, and membrane potential dynamics in live cells. Moreover, the modular design of these cationic fluorophores allows their conjugation to drugs, nanoparticles, or other bioactive molecules, facilitating targeted mitochondrial delivery. Ross et al. (2004) [124] also emphasized that the efficacy of mitochondrial accumulation depends on both the charge density and the lipophilicity of the cation, suggesting that these parameters can optimize targeting efficiency while minimizing cytotoxicity. Overall, lipophilic cation-based targeting remains a cornerstone in the design of mitochondria-specific probes, providing a versatile platform for bioimaging, diagnostics, and targeted therapeutics [124,128] (Figure 6).

Schematic illustration of carbon dot (CD)-based strategies for mitochondrial targeting and bioimaging. Two main approaches are highlighted: (i) TPP-conjugation, where lipophilic cations such as triphenylphosphonium (TPP) are covalently attached to CDs to promote mitochondrial accumulation via electrostatic attraction to the negative mitochondrial membrane potential; and (ii) intrinsic targeting, where CDs possess inherent mitochondrial affinity due to their surface chemistry and charge. Once localized within mitochondria, CDs enable fluorescence imaging and tracking, reactive oxygen species (ROS) sensing for monitoring oxidative stress, and theranostic applications combining diagnosis and therapy. Intrinsically targeted CDs can further support drug delivery and energy dynamics studies.

TPP-conjugated carbon dots (TPP-CDs) synthesized from urea and citric acid by hydrothermal method showed a diameter of 8.5 nm, displayed absorption and emission maxima at 340 nm and 425 nm and exhibited high biocompatibility and low cytotoxicity under incubation periods of 6–8 h. These CDs contained –NH_2_, –COOH, and –OH functional groups, which enabled covalent conjugation with TPP-COOH through amidation and two-photon confocal imaging demonstrated clear mitochondrial accumulation. Mitochondria serve as the primary intracellular source of reactive oxygen species (ROS) and increased mitochondrial ROS production has been implicated in multiple pathological conditions [129].

Consequently, the detection and localization of ROS within mitochondria are of significant importance. In this context Wu et al. (2017) developed TPP-functionalized carbon dots from o-phenylenediamine for the selective detection of peroxynitrite (ONOO^−^) [25].

These carbon dots exhibited characteristic absorption bands of amine-derived precursors. Upon exposure to ONOO^−^, fluorescence quenching occurred via photoinduced electron transfer, accompanied by the formation of benzotriazole structures through the reaction of surface o-diaminobenzene moieties with •NO_2_ generated during ONOO^−^ decomposition. Imaging of MCF-7 cells revealed reduced fluorescence following treatment with lipopolysaccharide and interferon-γ, confirming the suitability of the probe for intracellular peroxynitrite detection [25,130,131,132].

Mitochondria-targeted TPP-CDs have considered also in theranostic applications [133,134,135,136,137].

Notably, Gong et al. (2019) [127] reported the development of MitoCAT-g, a multifunctional mitochondria-targeted probe that acts both as an oxidative stress amplifier and a potential anticancer agent. The probe was synthesized using a three-step strategy: CDs were first prepared from citric acid and polyene polyamine, subsequently loaded with atomically dispersed gold, and finally conjugated with cinnamaldehyde to promote ROS generation and with TPP to achieve mitochondrial targeting. MitoCAT-g operates by depleting mitochondrial glutathione, thereby increasing ROS levels and inducing apoptosis [127]. For imaging and intracellular tracking, the probe was further labeled with fluorescein [127].

Despite their interesting applications, TPP and other cationic targeting moieties can exhibit dose-dependent toxicity and have been associated with hemolysis and adverse effects on the mononuclear phagocyte system. In fact TPP-CDs represent an interesting platform for mitochondrial targeting in bioimaging and nanomedicine, several limitations and potential safety concerns should be carefully considered. The strong mitochondrial accumulation driven by the lipophilic TPP^+^ moiety may disrupt mitochondrial membrane potential, impair respiratory chain function, and promote excessive ROS generation, ultimately leading to mitochondrial dysfunction and apoptosis, particularly in non-target healthy cells with high metabolic activity [25,129]. Consequently, despite their considerable potential, TPP-CDs remain at a preclinical stage, and comprehensive toxicological and pharmacokinetic evaluations are still required prior to clinical translation. These limitations highlight the need for alternative design strategies for production of CDs with an intrinsic ability to target mitochondria, thus avoiding the use of external lipophilic cations such as TPP^+^. In this approach, precursor engineering, heteroatom doping, and surface functionalization are exploited to modulate surface charge, lipophilicity, and molecular affinity toward mitochondrial membranes. Such intrinsically targeted CDs have shown preferential mitochondrial localization with improved biocompatibility, reduced nonspecific accumulation, and potentially lower mitochondrial toxicity [57,138,139,140].

In this context it is reported that CDs synthesized from mercaptosuccinic acid, ethylenediamine, and chitosan through a one-pot hydrothermal treatment showed a size of 2.1 ± 0.3 nm and are suitable for long-term mitochondrial imaging [141]

Inspired by this intrinsic targeting capability, Gao and Jiang et al. developed CDs that, not only localize to mitochondria, but also distinguish cancer cells from normal cells. These CDs were synthesized using glycerol and (3-aminopropyl)trimethoxysilane (APTMS) and exhibited an average diameter of 3.5 ± 0.5 nm, The green-emitting CDs showed efficient mitochondrial targeting across multiple cell lines with low cytotoxicity and cancerous cells stained with [138].

Another important aspect to consider is that for imaging applications, is desirable to use CDs with longer emission to reduced photodamage, greater tissue penetration, and minimal interference from autofluorescence. Geng et al. (2019) were the first to synthesize CDs that combined intrinsic mitochondrial targeting with tunable long-wavelength fluorescence [142].

Their retrosynthetic design employed m-aminophenol and citric acid, introducing a rhodamine-based luminescent center. The CDs exhibited bathochromic shifts when the substituent groups derived from m-aminophenol were varied. The positively charged rhodamine moiety facilitated mitochondrial accumulation by exploiting the negative membrane potential. In vivo studies in zebrafish confirmed low cytotoxicity and high biocompatibility, indicating strong potential for bioimaging and biodiagnostic applications [143,144].

Mitochondrial dysfunction is implicated in a wide range of diseases, including neurodegenerative disorders, inflammation, cardiac dysfunction, and diabetes. Therefore, monitoring mitochondrial behavior and microenvironmental changes is essential for understanding cellular fate and gaining deeper insight into the origin and progression of mitochondrial-related diseases [145].

## 6. Challenges and Future Directions

CDs have attracted significant attention due to their optical properties, low toxicity, and biocompatibility. They have potential applications in a variety of fields, including bioimaging, drug delivery, sensing, and optoelectronics. Despite their promising properties, the development of carbon dots is still in its nascent stages, with several challenges hindering their widespread application. The synthesis of carbon dots typically involves methods like hydrothermal or solvothermal processes, microwave-assisted methods, and laser ablation. However, these techniques are often complicated, time-consuming, and challenging to scale up for industrial production [146,147].

The variability in the size, shape, and fluorescence properties of CDs during synthesis poses a significant challenge for reproducibility and standardization. Achieving uniformity in particle size and surface chemistry is crucial for consistent performance. Surface passivation with functional groups (e.g., amino, carboxyl, and hydroxyl groups) is essential for tailoring the optical and chemical properties of CDs, and enhancing their solubility, stability, and biocompatibility [49,148].

Achieving precise control over surface functionalization while maintaining the intrinsic properties of the CDs is difficult. Moreover, the stability of functional groups under various environmental conditions is a concern for long-term applications. CDs exhibit fluorescence properties that depend on factors such as size, surface states, and synthetic conditions. The emission wavelength and intensity can be tuned by controlling these factors [149,150].

The fluorescence of CDs can be sensitive to environmental factors like pH, temperature, and solvent polarity, which could limit their practical applications. Additionally, improving the quantum yield and ensuring stable fluorescence over time remains a challenge. Carbon dots are generally considered to be non-toxic and biocompatible, making them promising candidates for biomedical applications [7,8,146].

However, comprehensive studies on the long-term effects and potential toxicity of CDs in vivo are still limited. A thorough understanding of the toxicological profiles of CDs is needed to ensure their safety for human use, especially for drug delivery and imaging purposes. Variations in synthesis methods and precursor materials can also affect their biocompatibility. The fluorescence mechanism of carbon dots is still not fully understood, and various models have been proposed, including surface state-related fluorescence and core-related fluorescence. There is a lack of consensus on how the fluorescence properties arise at the molecular level, which makes it challenging to design CDs with predictable and tunable properties [150,151].

Researchers are working on developing more efficient, scalable, and environmentally friendly synthesis methods that can produce uniform CDs with controlled properties. Green synthesis methods, using renewable resources or waste materials, hold promise for making the production process more sustainable [152].

Advancing surface functionalization techniques will be key to improving the versatility of carbon dots. Tailoring the surface chemistry to enhance targeting efficiency for drug delivery, bioimaging, and sensing is an important area of research. The development of multifunctional CDs, which combine imaging, therapeutic, and sensing capabilities, could open new avenues in theranostics. Future research will focus on improving the quantum yield, fluorescence stability, and tunability of CDs by optimizing their size, surface chemistry, and structural design. Advances in creating multi-colored and multi-emissive CDs will enhance their potential for multiplexed imaging and sensing applications [153].

The integration of carbon dots into complex devices such as sensors, optoelectronic components, and photonic devices is an exciting future direction. For biomedical applications, the combination of CDs with other nanomaterials, such as gold nanoparticles or polymeric nanoparticles, could improve their functionality in drug delivery and imaging. Comprehensive toxicity assessments, including long-term studies in vivo and environmental impact assessments will be crucial for the safe commercialization of CDs, especially in biomedical and consumer product applications. Standardized protocols for testing biocompatibility will help accelerate their adoption in clinical and industrial settings. Further theoretical studies and computational modeling are needed to gain a deeper understanding of the mechanisms behind the optical properties of CDs. Such studies could guide the design of new CDs with more predictable and tunable properties, accelerating their development for various applications [154].

Future challenges include designing CDs with intrinsic organelle targeting, long-wavelength emissions for in vivo applications, multifunctional theranostic capabilities, and standardized, scalable, and green production methods to ensure reproducibility, safety, and clinical applicability.

## 7. Conclusions

The synthesis of carbon dots is increasingly becoming more realistic, controllable, reproducible, and reliable. The size distribution is becoming increasingly narrower, moving toward stable, crystalline nanoparticles, along with their functionalization with various oxygen functional groups that enable photoluminescence in specific visible bands. Among these, laser ablation and other techniques are attracting increasing interest from researchers and applications.

Carbon dots hold significant potential for a wide range of applications, but overcoming the current challenges related to synthesis, surface functionalization, optical properties, and toxicity will be essential for their future success. Recent advances in green and sustainable synthesis methods offer the possibility of producing CDs with controlled size, surface chemistry, and optical properties, reducing environmental impact and improving scalability. With continued research and innovation, carbon dots could revolutionize fields such as medicine, environmental monitoring, and materials science.

In biomedical applications, multifunctional CDs capable of simultaneous imaging, therapeutic delivery, and sensing (theranostic CDs) represent a promising avenue for precision medicine. Their intrinsic targeting abilities, especially toward mitochondria or cancer cells, and the development of long-wavelength-emitting CDs, could enable deeper tissue imaging with minimal photodamage. The future of carbon dots is promising, but it requires a multidisciplinary approach to address these challenges and unlock their full potential [155,156,157].

Standardized protocols for toxicity assessment, combined with computational modeling to predict fluorescence behavior and targeting efficiency, will accelerate the translation of CDs from bench to bedside. Moreover, integrating CDs with other nanomaterials, optimizing their biocompatibility, and advancing sustainable production will be key strategies to fully realize their applications in theranostics, biosensing, and optoelectronic devices.

## Data Availability

No new data were created or analyzed in this study. Data sharing is not applicable to this article.

## Abbreviations

CDs	Carbon Dots
ROS	Reactive Oxygen Species
ATP	Adenosine triphosphate
OXPHOS	Oxidative Phosphorylation
ETC	Electron Transport Chain
TPP	Triphenylphosphonium
